# Level of physical activity among urban adults and the socio-demographic correlates: a population-based cross-sectional study using the global physical activity questionnaire

**DOI:** 10.1186/s12889-019-7465-y

**Published:** 2019-08-22

**Authors:** Melkamu Merid Mengesha, Hirbo Shore Roba, Behailu Hawulte Ayele, Addisu Shunu Beyene

**Affiliations:** 1 Department of Epidemiology and Biostatistics, College of Health and Medical Sciences, School of Public Health, Haramaya University, P.O. Box 235, Harar, Ethiopia; 2 Department of Public Health and Health Policy, College of Health and Medical Sciences, School of Public Health, Haramaya University, Harar, Ethiopia; 3 Department of Environmental Health Sciences, College of Health and Medical Sciences, Haramaya University, Harar, Ethiopia; 4 Research Centre for Generational Health and Ageing, School of Medicine and Public Health, Faculty of Health and Medicine, University of Newcastle, Newcastle, Australia

**Keywords:** GPAQ, Level of physical activity, Correlates, Adult, Dire Dawa

## Abstract

**Background:**

Globally, in 2016, 23.3% of adult populations were physically inactive, and it accounts for 9% of the global premature mortality. However, evidence on the level of physical activity was limited in resource-poor settings. This study, therefore, assessed the adult’s level of physical activity and its correlates among the urban population in Dire Dawa, eastern Ethiopia.

**Methods:**

A total of 872 randomly selected adults of age 25–64 years in Dire Dawa city, eastern Ethiopia, are included in this analysis. The Global Physical Activity Questionnaire (GPAQ) is used to measure physical activity. Individuals are considered physically active when they achieved metabolic equivalent tasks (MET) minutes of 600 or more per week, and otherwise inactive. A binary logistic regression is used to identify the correlates of physical activity.

**Results:**

54.9% (95% confidence interval (CI) 51.6 to 58.2) of adults were physically active, with a higher proportion of men being physically active than women, 63.9% (95% CI 58.1 to 69.3) versus 50.6% (95% CI 46.5 to 54.6). Among the adults who reported doing physical activity, the highest domain-specific contribution to the total physical activity was from activities at workplaces, and leisure-time activities contributed the least. The proportion of adults who engaged in a high-level physical activity was 37.0% (95% CI 33.9 to 40.3). Male adults were 1.45 times (aOR (adjusted odds ratio) =1.45; 95% CI 1.05 to 1.99) more likely to achieve the recommended level of physical activity. In contrary, unemployed adults (aOR = 0.51; 95% CI 0.35 to 0.75) were less likely to perform the recommended level of physical activity to accrue health benefits.

**Conclusion:**

Interventions aimed at promoting physical activity should target unemployed and female adults. We recommend further study to explore the contextual factors that hinder physical activity in the study setting.

**Electronic supplementary material:**

The online version of this article (10.1186/s12889-019-7465-y) contains supplementary material, which is available to authorized users.

## Background

Insufficient physical activity is the fourth leading cause of death worldwide [1]. The World Health Organization (WHO) defines the level of physical activity less than 600 metabolic equivalent tasks (MET) minutes per week as not sufficient to produce health benefits [2]. Physical inactivity is causally associated with coronary heart diseases, breast cancer, and colon cancer [3]. Additionally, it accounts for 9% of the global premature mortality [3]. In terms of the burden on the economy, deaths due to physical inactivity contribute to $13.7 billion in productivity losses. Additionally, regarding its impact on an individual’s quality of life, physical inactivity accounts for 13.4 million disability-adjusted life years (DALYs) worldwide [4].

Globally, in 2016, the prevalence of physical inactivity among adult populations was 23.3% [5]. A review by Guthold R et al. in 22 African countries reported that 20% of adults were physically inactive [6]. In Ethiopia, the WHO STEPwise approach to Surveillance (STEPS) survey report for Addis Ababa showed a slight increase in the proportion of physically inactive adults between 2006, and 2015, 26% versus 28.8% [7, 8].

Physical inactivity is a modifiable risk factor for NCD, and being physically active has several benefits for health. The population attributable fraction (PAF) of physical inactivity for coronary heart disease, type 2 diabetes, breast cancer, colon cancer, and all-cause mortality was 5.8, 7.2, 10.1, 10.4, and 9.4%, respectively [3]. A study by Huerta et al. reported that physical activity at workplaces and household chores were strongly associated with a reduced overall and cause-specific mortality in women and to lower cancer mortality in men [9]. There are also strong pieces of evidence that physical activity increases cardiorespiratory and muscular fitness, healthier body mass and composition, improved bone health, increased functional health, and improved cognitive function [3, 5]. A meta-analysis, on the dose-response relationship of physical activity and mortality, reported that higher levels of total and domain-specific physical activities reduce all-cause mortality [10]. Despite that physical activity has several benefits, the population around the world continue to suffer from the pandemic of physical inactivity [1, 3, 9]. Attempts to respond to this pandemic has led countries including physical activity in their national policies, even though, this response is too slow and ineffective [5].

Research evidence reported correlates of physical activity at the individual, interpersonal, and environmental levels [11–13]. For instance, Bauman et al. reported that male gender, level of education, self-efficacy, and social support were positive correlates of physical activity. Whereas, age, overweight, and perceived effort are inversely correlated [11]. The environmental correlates of total physical activity among adults include access to recreation facilities and locations, transportation environment, and aesthetics [11, 14, 15]. A study examining physical inactivity in India reported that there was no statistically significant difference in the proportion of the physically inactive population, based on gender, or age groups [16]. Despite several studies are conducted in high-income or upper-middle-income countries, on the correlates of physical activity, there was limited evidence in low-income countries to guide locally appropriate interventions [11].

In Ethiopia, the STEPS surveys conducted in 2006 in Addis Ababa, and at the national level in 2015, did not report the correlates of physical activity [7, 8]. Therefore, this study aimed to assess the level of physical activity and its correlates among the urban population in Dire Dawa, eastern Ethiopia. In this study, we collected data from a large representative sample compared to the sub-national sample for Dire Dawa in the national STEPS survey [7].

## Methods

### Study setting and design

A population-based cross-sectional study was conducted from June 01/2017 to June 21/2017 in Dire Dawa city, eastern Ethiopia. The city has 4 Keftegnas (districts) and 9 urban kebeles (sub-district, the lowest administrative unit). Located at 515 km to the southeast of Addis Ababa, Dire Dawa city is the capital of the Dire Dawa Administrative Council (DDAC) which has an estimated urban population of 252, 279 [17]. Dire Dawa lies between 1000, and 2000 m above the sea level. It has an average monthly temperature of 24.8 degree Celsius and an average annual rainfall of 604 mm [18].

### Participants

Adults aged 25–64 years, who lived in Dire Dawa city for at least six months before the survey, are included in this study. In each household, an adult resident was identified, and only one was randomly selected to participate in the study when there was more than one adult in that household. Excluded from the study were pregnant women, bedridden adults, and those who had a disability that limits activity.

### Sample size

This study used the sample calculated for a study on metabolic syndrome (unpublished) in the study setting. A single population proportion formula was used to calculate the sample size for the original study. The sample size is calculated using the OpenEpi v 2.3 software [19]. The assumptions used to calculate the sample size were the prevalence of metabolic syndrome, *P* = 17% [20]; margin of error, d = 3%; a design effect of 1.5 to compensate for a random error due to a multi-stage sampling, and 95% level of confidence. Accordingly, the minimum calculated sample size was 903. Of the total 903 samples, 872 subjects who completed the survey were available for this analysis.

### Sampling technique

Multi-stage sampling was used to select the sub-districts, primary sampling units (households), and the study units (adults, 25–64 years). From the 9 urban sub-districts in the study setting, five were randomly selected. A sampling frame was created using a list of households obtained from the Dire Dawa city sub-district administration. The study sample was then proportionally allocated to each of the selected sub-districts based on their size. Systematic random sampling was used to select the primary sampling units. When there was more than one eligible adult in a selected household, one was randomly selected.

### Data collection

The WHO STEPS instrument was used to collect the data [21]. The English version of the STEPS instrument was translated into the two widely spoken local languages in the study setting, Amharic and Afan Oromo, and then, it was back-translated to English to check the consistency. When inconsistency occurred, based on consensus, corrections are made as appropriate. Before the actual data collection, a pretest of the instrument was conducted using the local translated version. For this analysis, we considered variables on demographic information, behavioral measurements (physical activity), and physical measurements (height and weight). Weight is measured using a standard portable digital scale. Stadiometer was used to measure height, and the results are recorded to the nearest 0.5 cm. Demographic information collected includes age, sex, education, ethnicity, marital status, and occupation. The occupation variable assessed participant’s main work status over the past 12 months. Adults participated in this study are said to be unemployed if she or he was not in employment, not engaged in any activity to produce goods or services for pay or profit during the 7 days before the survey, or in the 12 months before the survey. Research assistants who completed at least diploma level of education and had a health background collected the data and the data collection activity were supervised daily. Three days orientation was given to research data assistants on the study protocol including its objective, data collection methods, STEPS survey procedure, and also on data quality.

### Definitions and measurements

The global physical activity questionnaire version-2 (GPAQ-2) was used to measure physical activity [22]. It has a total of 16 questions (P1-P16) that assess sedentary behavior and three domains in which physical activity is performed: work, transport, and leisure-time physical activity. Study participants were asked if they had engaged in vigorous and moderate work and leisure-time activities continuously for at least 10 min. Transport related activities include only moderate-intensity activities worked out continuously for at least 10 min. Participants responding affirmatively of their engagement in a specific activity were asked about the number of days engaging in each activity in a typical week, and the time spent in each activity in a typical day. The responses to the frequency and duration questions are used to calculate the total amount of time a person spent doing physical activity or MET minutes per week. Vigorous-intensity activity is defined as an activity that makes an individual breathe much harder than normal, and a moderate-intensity activity makes an individual breathe somewhat harder than normal. We used the generic GPAQ show cards to aid in obtaining consistent and valid measurements [22]. In the GPAQ, sedentary behavior was assessed through the question “How much time do you usually spend sitting or reclining on a typical day?”(Additional file 1: Table S1).

For adults aged 18–64 years, the WHO global recommendation on physical activity for health is to do at least 150 min of moderate-intensity physical activity throughout the week; or 75 min of vigorous-intensity physical activity throughout the week; or an equivalent combination of moderate- and vigorous-intensity activity accumulating at least 600 MET-minutes per week [22]. In our study, we used this cut-off to define physically active (achieved 600 or more MET-minutes per week) versus inactive adults (achieving less than 600 MET-minutes per week).

We also characterized adult’s physical activity using cut-offs into high (achieving a minimum of at least 3000 MET-minutes per week from any combination of walking, moderate-or vigorous-intensity activities or accumulating 1500 MET-minutes per week from vigorous-intensity activity), moderate (a person not meeting the criteria for the high category, but achieved a minimum of at least 600 MET-minutes per week from any combination of walking, moderate-or vigorous-intensity activities) and low (a person not meeting any of the above criteria. This included those reporting no activity or some, but not enough to meet the high and moderate categories) [23].

### Data processing and analysis

The data were entered into EpiData version 3.0 and then exported to STATA version 14.2 statistical software for management and analysis. We followed the GPAQ-2 data processing and analysis protocol, where all the three domains of physical activity are cleaned as a combined set, to produce estimates on physical activity [22]. Issues of missing values were handled using multiple imputations by chained equations (MICE) method [24] for five variables which accounted for 11.5% of missing values if a complete case analysis would have been considered. The variables imputed include ethnicity (48 values), years in school (33 values), height (27 values), weight (13 values) and age (12 values). Before imputation, we checked the pattern of missing, and the Little’s missing completely at random (MCAR) test in STATA supported that all the variables were MCAR (chi-square (df) =159.55 (172), *p*-value = 0.743).

In each of the three domains of physical activity in the GPAQ, responses to questions on the frequency and duration of physical activity are used to convert the total time spent on activity to energy expenditure, the MET-minutes [22]. A MET is the ratio of specific physical activity metabolic rates to the resting metabolic rate, with one MET defined as the energy cost of sitting quietly which is equivalent to l kcal/kg/hour [23]. Compared to the energy expenditure when seated quietly, a person’s caloric consumption is four times higher when being moderately active, and eight times higher when being vigorously active. Accordingly, moderate-intensity activities during work, transport, and leisure-time are assigned a value of 4 METs; and vigorous-intensity activities are assigned a value of 8 METs. Hence, to convert a vigorous-intensity activity for 75-min in a week into MET-minutes, multiply 75-min by 8-METs, which equals 600 MET-minutes per week. Consequently, the total physical activity score is computed as the sum of all MET-minutes per week from moderate- to vigorous-intensity activities performed at workplaces, during transport, and leisure-time [22]. The total physical activity of ≥600 MET-minutes per week denotes adequate physical activity or being physically active, and an inadequate level of physical activity or physical inactivity was represented by < 600 MET-minutes per week. Adequate physical activity was coded ‘1’ and an inadequate level of physical activity was coded ‘0’. The results are summarized in tables, graphs and also presented in text. We used the two-sample Wilcoxon rank-sum (Mann-Whitney) test to see the relationship between two variables, and the binary logistic regression to identify correlates of physical activity. For all statistical tests, significance was defined as *p*-value< 0.05.

### Ethical clearance

Ethical clearance was obtained from the Haramaya University College of Health and Medical Sciences’ Institutional Health Research Ethics Review Committee (IHRERC), with an approval number, IHRERC/003/2016. Additionally, to conduct the field activity in Dire Dawa, a letter of permission was obtained from the DDAC. Informed written consent was sought beforehand to obtain data from each of the participants. Confidentiality of the information collected is maintained by keeping the anonymity of individual participants at all levels.

## Results

### Socio-demographic characteristics

A total of 872 subjects participated in this study, and more than half (67.2%) of them were females. The majority, 64% of the study participants, were below the age of 45 years with a mean age of 40.4 years. Fifty-two percent of the respondents either completed primary education or had no formal education. Unemployed participants constituted 45.0% of the sample, while 27.0% were office work employees. Currently married adults constituted 67.0% of the sample (Table 1).

**Table 1:** Socio-demographic characteristics of adults (25–64 years) in Dire Dawa city, eastern Ethiopia, 2017

Variables		Frequency, *n* = 872	Percent (%)
Age, mean (SD)	40.4 (13.0)		
Age	25–29	231	26.5
	30–44	326	37.4
	45–54	124	14.2
	55–64	191	21.9
Sex	Male	285	32.7
	Female	587	67.3
Education	No formal education	125	14.3
	Primary (1–8 grade)	327	37.5
	Secondary (9–12 grade)	316	36.2
	College diploma and above	104	11.9
Marital status	Currently married	582	66.7
	Never married	173	19.8
	Divorced/Widower	117	13.4
Ethnicity	Oromo	302	34.6
	Amhara	428	49.1
	Others^a^	142	16.3
Occupation	Employed office work	234	26.8
	Merchant	134	15.4
	Unemployed	396	45.4
	Others§	108	12.4

^a^Ethio-Somali, Harari, Tigraway, Guraghe, Wolayta, Silte, Afar; §housewife, pensioner, janitor, and daily laborer; *SD* = standard deviation

### Self-reported physical activity by specific domains

**Fig. 1:**
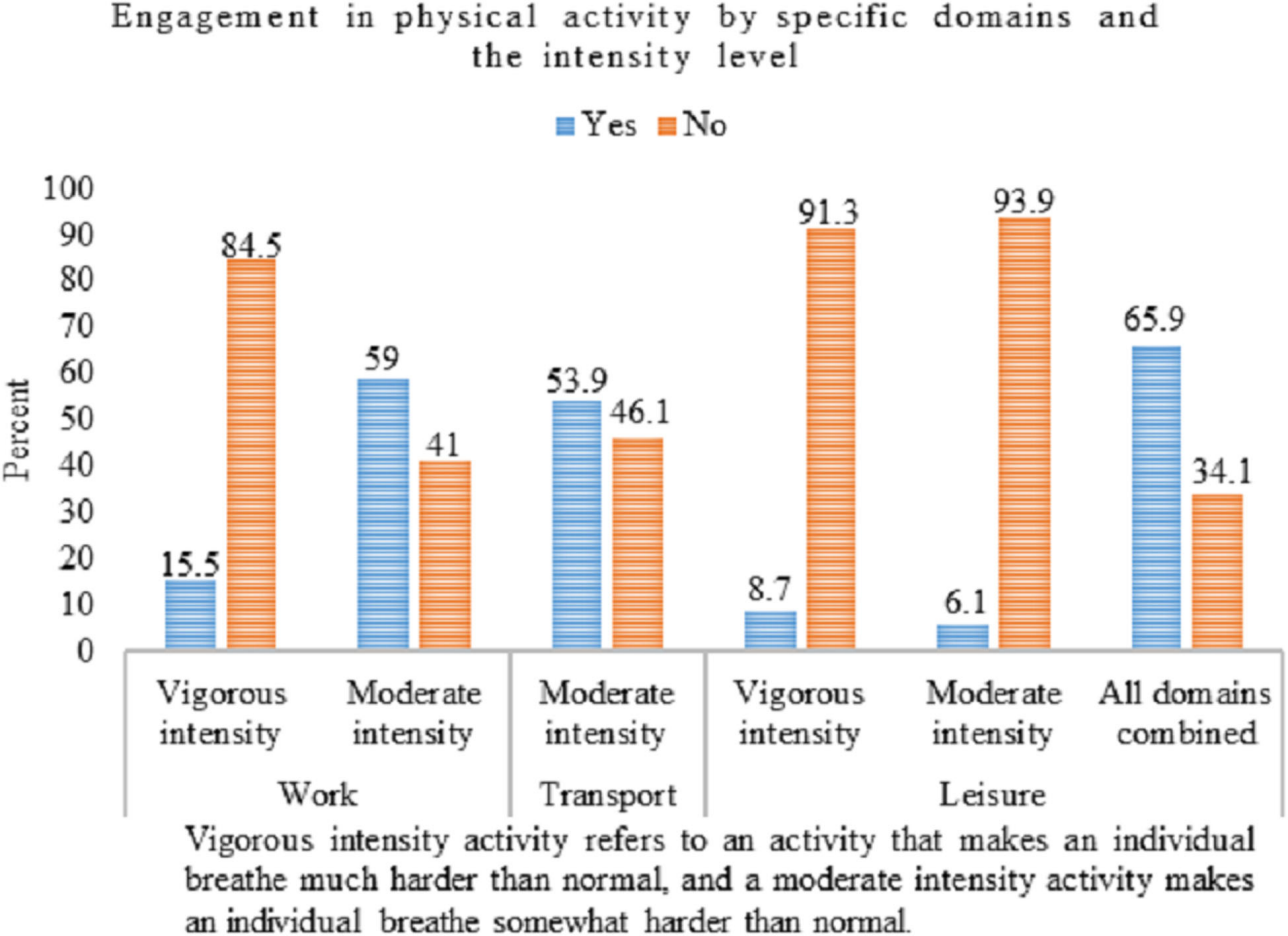
Proportion of adults (25–64 years) who engage in physical activity by specific domains and intensity level in Dire Dawa city, eastern Ethiopia, 2017

Of the total study participants, 34.1% reported no physical activity that lasted for at least 10-min continuously in any of the three physical activity domains. Whereas, out of the 65.9% who reported doing physical activity for at least 10-min continuously, 59.0% reported moderate-intensity physical activity at workplaces and 53.9% reported activity during travel to and from places. These are the common physical activity in the study setting. Vigorous- and moderate-intensity leisure-time activities were the least commonly practiced among adults (Fig. 1).

Compared to the other physical activity domains, activities at the workplaces had the highest median percent contribution to the total physical activity, 93.0%, with no statistically significant difference between males and females. In contrast, the median percent contribution of activities during leisure-time to the total physical activity was null in the study setting (Table 2).

**Table 2:** Self-reported physical activity in a typical week among adults (25–64 years) in Dire Dawa city, eastern Ethiopia, 2017

Physical activity measures	Both sexes, *n* = 872; (95% CI)	Male, *n* = 285; 95% CI	Female, *n* = 587; 95% CI	Pearson’s Chi2 (df)	*P*-value
Total MET minutes per week, median (IQR)^d^	5040 (672, 11,520)	10,080 (1680, 14,400)	2580 (672, 10,080)	Z = 5.8	< 0.001^c^
Domain specific median percent contribution to the total physical activity					
Work (median % (IQR))^d^	93.0 (0, 100)	90.3 (0, 100)	95.4 (0, 100)	Z = 0.4	0.659^c^
Transport (median % (IQR))^d^	2.2 (0, 100)	1.6 (0, 26.9)	4.1 (0, 100)	Z = -2.8	0.004^c^
Leisure (median % (IQR))^d^	0.0	0.0 (0, 3.4)	0.0	Z = 8.7	< 0.001^c^
The proportion of participants within a specific level of intensity of physical activity					
Vigorous physical activity, ≥75 min/week^d^	21.6 (18.4, 25.1)	39.3 (32.7, 46.3)	12.0 (9.1, 15.8)	57.5 (1)	< 0.001
Moderate physical activity, ≥150 min/week^d^	77.9 (74.3, 81.1)	83.1 (77.2, 87.7)	75.1 (70.4, 79.3)	4.8 (1)	0.028
Both moderate and vigorous physical activity^d^	16.2 (13.4, 19.4)	31.8 (25.7, 38.7)	7.8 (5.4, 11.0)	55.9 (1)	< 0.001
The proportion of participants within a level physical activity category^b^					
High	37.0 (33.9, 40.3)	47.4 (41.6, 53.2)	32.0 (28.4, 35.9)	20.1 (1)	< 0.001
Moderate	17.9 (15.5, 20.6)	16.5 (12.6, 21.3)	18.6 (15.6, 21.9)		
Low	45.1 (41.8, 48.4)	36.1 (30.7, 41.9)	49.4 (45.4, 53.5)		
Median time (IQR) spent sitting^a^	300 (180, 480)	240 (120, 420)	300 (240, 480)	Z = -3.7	< 0.001^c^

^a^Duration measured in minutes

^b^low: < 600 MET-minutes per week; moderate: 600 to < 3000 MET-minutes per week, and high: ≥3000 MET-minutes per week

^c^Two-sample Wilcoxon rank-sum (Mann-Whitney) test; *IQR* = Inter quartile range, *CI* confidence interval

^d^computation did not include those who reported no activity (*N* = 297: Male = 84, Female = 213)

Approximately 22% (95% CI 18.4 to 25.1) of the adults did vigorous-intensity activity for 75-min or more per week. The proportion of men who did vigorous-intensity activity were more than three-fold higher compared to women, 39.3% (95% CI 32.7 to 46.3) versus 12.0% (95% CI 9.1 to 15.8), and the difference was statistically significant. Similarly, a significantly higher proportion of men were engaged in either a moderate-intensity activity or a vigorous-intensity activity. Of the adults who reported doing physical activity, 16.2% (95% CI 13.4 to 19.4) did both vigorous and moderate-intensity activity, and this too was significantly higher among men. The proportion of adults who did high-level physical activity was 37.0% (95% CI 33.9 to 40.3). Regarding sedentary activities, women had significantly higher median time spent in sitting compared to men, 300 min per week versus 240 min per week (Table 2).

### Physical activity level in the study setting against the WHO recommendation

**Fig. 2:**
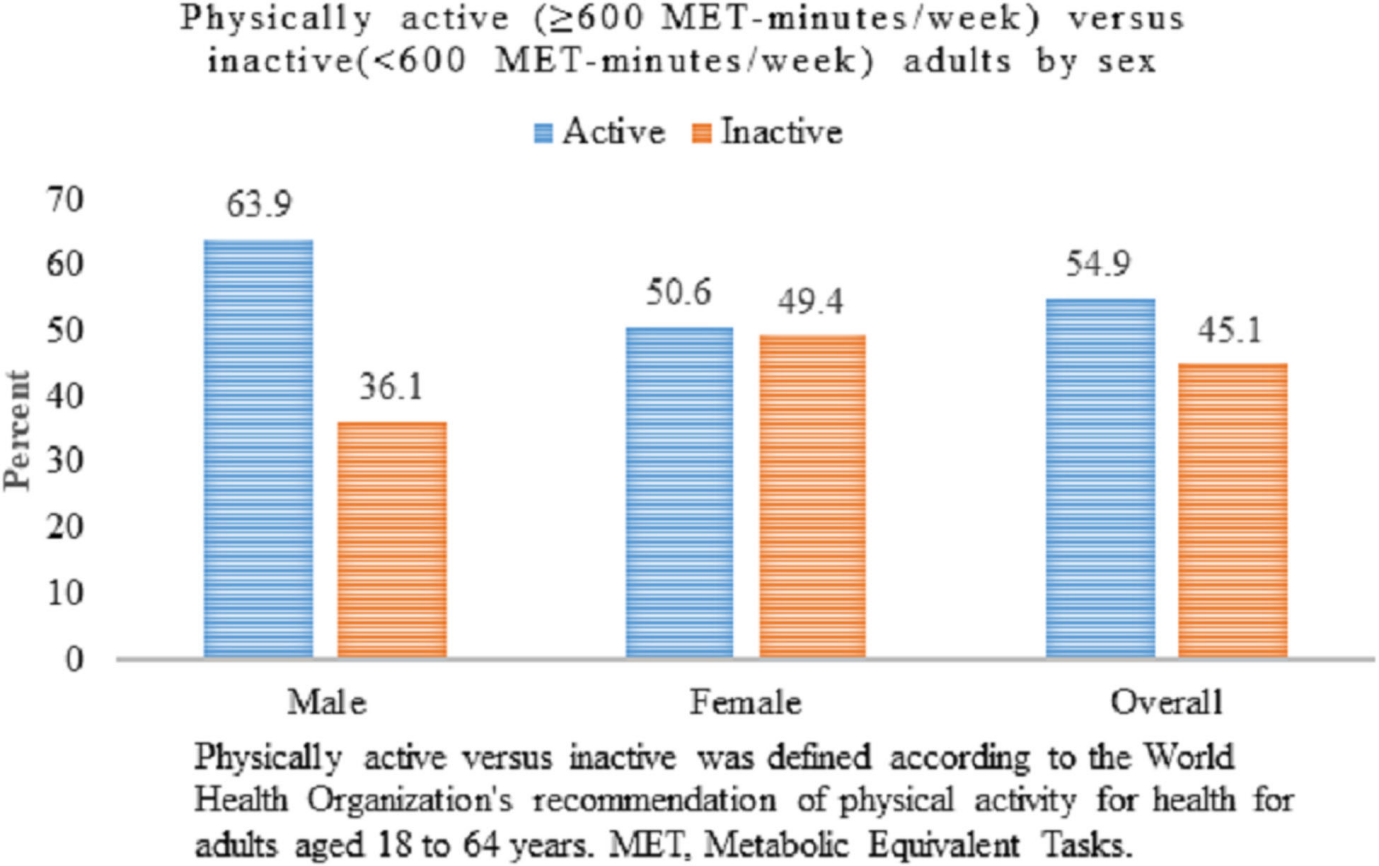
Proportion of adults (25–64 years) who achieved the WHO global recommendations on physical activity for health by gender in Dire Dawa city, eastern Ethiopia, 2017

The proportion of physically active adults in this study was 54.9% (95% CI 51.6 to 58.2), with a higher proportion of men being physically active than women, 63.9% (95% CI 58.1 to 69.3) versus 50.6% (95% CI 46.5 to 54.6) (Fig. 2).

When the participant’s level of physical activity was disaggregated by the WHO global recommendation on physical activity for health, females who achieved the recommended level were over 10% lower than those who did not, 62.0% versus 73.8%. Similarly, 39.5% of unemployed adults achieved the recommended level, and 52.7% of the same group did not. In contrast, a positive difference between the proportion of physically active and physically inactive adults are observed among men, among those who had primary education, office employees, and among those who had a normal BMI (Table 3).

**Table 3:** Distribution of adults (25–64 years) who achieved and did not achieve the World Health Organization (WHO) recommendations on physical activity for health in Dire Dawa city, eastern Ethiopia, 2017

Variables	All groups, *n* = 872	Proportion of adults by the WHO global recommendations on physical activity for health^c^	
		Achieved the recommended level, *n* = 479; % (n)	Did not achieve the recommended level, *n* = 393; % (n)
Age			
25–29	231	28.0 (134)	24.7 (97)
30–34	326	38.4 (184)	36.1 (142)
45–54	124	13.6 (65)	15.0 (59)
55–64	191	20.0 (96)	24.2 (95)
Sex			
Male	285	38.0 (182)	26.2 (103)
Female	587	62.0 (297)	73.8 (290)
Education			
No formal education	125	11.9 (57)	17.3 (68)
Primary education	327	40.1 (192)	34.4 (135)
Secondary education	316	35.7 (171)	36.9 (145)
College and above	104	12.3 (59)	11.5 (45)
Marital status			
Married	582	65.8 (315)	67.9 (267)
Not married	173	21.9 (105)	17.3 (68)
Divorced/widowed	117	12.3 (59)	14.8 (58)
Occupation			
Office employee	234	31.1 (149)	21.6 (85)
Merchant	134	15.7 (75)	15.0 (59)
Unemployed	396	39.5 (189)	52.7 (207)
Other^b^	108	13.8 (66)	10.7 (42)
Ethnicity			
Oromo	302	35.1 (168)	34.0 (134)
Amhara	428	47.4 (227)	51.2 (201)
Others^a^	142	17.5 (84)	14.8 (58)
Body mass index			
Underweight (< 18.5 Kg/m^2^)	66	7.9 (38)	7.1 (28)
Normal (18.5–24.99 Kg/m^2^)	472	57.0 (273)	50.6 (199)
Overweight (≥25.0 Kg/m^2^)	334	35.1 (168)	42.2 (166)

^a^Ethio-Somali, Harari, Guraghe, Tigraway, Wolayta, Silte, Afar

^b^housewife, pensioner, janitor, daily laborer

^c^Adults, 18–64 years, should accumulate at least 600 MET-minutes of physical activity per week (2); WHO, World Health Organization

### Factors associated with physical activity status

In the bivariate binary logistic regression being male, primary education attendance and those who had a normal BMI had a positive and significant association with being physically active.

In contrast, unemployed adults were less likely to be physically active. Compared to the office employed adults, unemployed adults were 48% less likely to be physically active (Table 4).

**Table 4:** Factors associated with doing physical activity of 600 MET-minutes per week or more among adults (25–64 years) in Dire Dawa city, eastern Ethiopia, 2017

Variables	cOR, (95% CI)	*p*-value	aOR, (95% CI)	*p*-value
Age				
55–64	Ref.		Ref.	
25–29	1.34 (0.91, 1.98)	0.134	1.33 (0.84, 2.10)	0.228
30–44	1.27 (0.89, 1.82)	0.187	1.22 (0.81, 1.82)	0.342
45–54	1.10 (0.70, 1.74)	0.675	1.08 (0.66, 1.74)	0.766
Sex				
Female	Ref.		Ref.	
Male	1.73 (1.29, 2.31)	< 0.001	1.45 (1.05, 1.99)	0.024
Education				
No formal education	Ref.		Ref.	
College diploma and above	1.52 (0.89, 2.60)	0.126	0.72 (0.38, 1.36)	0.308
Secondary (9–12 grade)	1.40 (0.91, 2.14)	0.125	0.88 (0.53, 1.46)	0.630
Primary (1–8 grade)	1.72 (1.12, 2.64)	0.012	1.35 (0.85, 2.15)	0.206
Marital status				
Married	Ref.		Ref.	
Never married	2.31 (0.93, 1.85)	0.127	1.26 (0.86, 1.86)	0.237
Divorced/widowed	0.86 (0.58, 1.28)	0.465	0.92 (0.61, 1.40)	0.704
Occupation				
Employed office work	Ref.		Ref.	
Merchant	0.73 (0.47, 1.12)	0.662	0.67 (0.42, 1.06)	0.087
Unemployed	0.52 (0.37, 0.73)	< 0.001	0.51 (0.35, 0.75)	0.001
Other^b^	0.90 (0.56, 1.43)	0.648		
Ethnicity				
Oromo	Ref.			
Amhara	0.87 (0.64, 1.19)	0.384		
Other^a^	1.15 (0.76, 1.73)	0.513		
BMI				
> =25.0 kg/m^2^	Ref.		Ref.	
< 18.5 kg/m^2^	1.39 (0.81, 2.38)	0.227	1.47 (0.85, 2.56)	0.170
18.5–24.99 kg/m^2^	1.35 (1.02, 1.80)	0.039	1.25 (0.93, 1.68)	0.142

^a^Ethio-Somali, Harari, Guraghe,Tigraway Wolayta, Silte, Afar

^b^housewife, pensioner, janitor, daily laborer; cOR, crude odds ratio; aOR, adjusted odds ration; CI, confidence interval

In the multivariable binary logistic regression, however, the positive association of adults who had normal BMI and those who had a primary school attendance with achieving physical activity of 600 MET-minutes per week or more was no longer significant. On the other hand, unemployment status and being male remained statistically significant. Adult males were 45% more likely to be physically active compared to women. Compared to office employed adults, those who were unemployed had an inverse association of doing the recommended level of physical activity for health (Table 4).

## Discussion

The proportion of physically active adults in this study was 54.9%. This study also found that unemployment status had a negative association with being physically active. In contrast, male gender had a positive association with being physically active.

Several multi-country studies that used the GPAQ to assess physical activity reported that level of physical inactivity, performing less than 600 MET-minutes per week [2], greatly varies from country to country, and also at the sub-national level [6, 25]. For example, Guthold et al. reported the proportion of physically active adults in 22 African countries, with the lowest value being in Mali (46.8%) and the highest in Mozambique (95.6%) [6]. A systematic review by Shahara et al. reported the proportion of physical inactivity ranging from 20.1% in Comoros to 67.6% in Saudi Arabia, and also in three subnational samples, the proportion ranged from 40.7% in Algeria to 51.3% in Mauritania, and 86.8% in Sudan [25]. The proportion of physically inactive adults in the current study setting was 45.1%, which is higher compared to the study from Mozambique or Comoros, but it is lower compared to other studies in Mali, Sudan, Mauritania, and Saudi Arabia [6, 25]. However, it is worth to note that our study was conducted at a sub-national level, and hence the level of physical inactivity may seem higher compared to studies that reported estimates at the national level [6, 13, 25].

In this study, physical inactivity was two times higher compared to that of the 2015 national STEPS survey specifically reported for Dire Dawa, 19.7% [7]. Two reasons could explain the observed difference: Firstly, the 2015 national STEPS survey included both urban and rural population of the DDAC, and this mix of a relatively physically active rural population as noted in other studies [13, 14] in the estimation might have masked the true level of physical inactivity in the urban Dire Dawa. Secondly, disproportionately more women were included in our study, and this may have inflated the level of physical inactivity as research evidence consistently reported women to be physically inactive than men [11, 13, 14]. Furthermore, the climate of Dire Dawa is hot [18], and this may have contributed to the high-level physical inactivity as different studies reported the influence of the built environment on physical activity [26]. Four out of every ten persons were physically inactive in the present study. This finding suggests the need to promote physical activity, which in turn requires a multi-sectoral, and multi-disciplinary public health response [1, 27]. With a high-level of physical inactivity, there is an increased risk of poor health outcome as several studies reported that physical inactivity is causally associated with deaths from non-communicable diseases and all-cause mortality [9, 10, 28, 29].

In the study setting, as in other low-and-middle-income countries (LMICs) [6, 13, 30], physical activity at workplaces and during transport contributed a significant share to the total physical activity. Due to a very low motor vehicle ownership, 8 motor vehicles per 1000 people in Ethiopia [31], most people walk to get to and from places. We also hypothesize that, in the study setting, people engage in activities that are labor-intensive during household chores or at workplaces due to low availability and access to technological devices that help them to get their work done with a low energy expedition. However, with a growth in the economy coupled with rapid urbanization, the current level of domain-specific contributions of physical activity at workplaces, and during transport in the study setting is expected to decrease as noted elsewhere [32, 33]. Therefore, to sustainably improve population-level physical activity and contribute towards reducing the global physical inactivity by 10% by 2025 [34], emphasis should be given to a systems approach that focuses on populations and the complex interactions among the correlates of physical inactivity including environmental factors [1, 27, 35, 36].

The contribution of leisure-time physical activity to the total physical activity in the present study was very low as in other similar settings [14, 30]. For example, in a study in Mozambique, Padrao et al. reported that leisure-time physical activity by women of age 35 to 44 years constituted only 2.2% of the total physical activity and 18.9% by men of age 25–34 years [14]. Even though there was a significant difference between men and women regarding the average contribution of leisure-time physical activity to the total physical activity in the study setting, it was very low compared to the study reported in Mozambique. Research evidence indicated that individuals from a higher socioeconomic status were likely to be contributing to a higher level of leisure-time physical activity [37]. However, no difference was reported in the physical activity domains other than the leisure-time physical activity domain [37].

For additional health benefits, research evidence suggests that adults should increase the intensity of physical activity well beyond the minimum recommended level, 600 MET-minutes per week [10, 29]. For example, compared to no physical activity, adult’s activity level of 600 MET-minutes per week lowers the risk of diabetes by 2%, and an increase of this level to 3600 MET-minutes per week reduces the risk by an additional 19% [29]. In our study setting, only 37.0% of the adults did a high-level total physical activity, and it was significantly different between men and women: 47.6% for men versus 31.9% for women. This finding is very low compared to a study reported 83.2% for urban women and 78.9% for urban men in Mozambique [14], but higher compared to the study in Bangladesh, 19.5% [13]. In addition to lowering the risk to non-communicable diseases, high-level physical activity was also reported to eliminate the risk of mortality associated with sitting time [28]. In the present study, due to 4-h or more sitting time coupled with a lower proportion of high-level physical activity, a significant risk of poor health outcome remains a challenge. For example, a meta-analysis reported a 27% increased risk of mortality among adults who sat less than 4-h per day and were in the lowest activity quintile [28].

In this study, we found that unemployment and male gender were significantly associated with physical activity. Consistent with previous reports [2, 16, 38, 39], unemployed adults were less likely to perform the recommended level of physical activity for health. We hypothesize that unemployed adults were likely to be in low socioeconomic status, and have poor access to resources for participation in physical activity [40]. A study on physical activity among Nepalese adults, however, reported that self- or government-employed adults were physically inactive [30]. As reported in previous studies, male adults were physically active compared to women [12, 30, 38, 41]. However, consistent individual-level correlates of physical activity in previous studies including age and level of education [14, 30] were not significantly associated with physical activity in our study. This may be due to a relative influence of the built environment over the individual factors in the study setting such as the hot climate as noted elsewhere [25, 26]. However, an explanation for this needs further investigation to assess contextual factors in greater depth.

The strengths of this study include that we used the GPAQ, and followed the GPAQ analysis protocol [22], which we believe allows comparison of findings from different settings. As an aid for consistency of physical activity measurement, we used the GPAQ showcards [22] and reported findings on all the three domain-specific physical activity (work, during transport to and from places, and leisure). However, this study is not without limitation, and hence worth to mention to help readers interpret the findings with caution. Firstly, the study unintentionally included more women, and this might have biased the overall estimate of physical activity in the study setting. Secondly, we did not assess the social, cultural, and environmental factors that may influence physical activity, and the authors would like to suggest that future studies consider the effect of these factors on physical activity. Finally, the cross-sectional nature of our study did not allow us to see the temporal relation between the assessed factors and physical activity.

## Conclusion

This study presented findings on the most overlooked public health problem in a resource-poor setting [11], yet a global pandemic that drives the burden of non-communicable diseases and premature mortality [11]. In the study setting, 54.9% of urban adults were physically active, and women were less active than men. Workplace physical activity and activity during transport contributed a significant share to the total weekly physical activity. To further improve the proportion of physically active people in the study setting, promoting physical activity at workplaces, improving trails for commuting, and targeted expansion of facilities that satisfy the needs of unemployed adults and women will be beneficial.

## Additional file

Additional file 1: **Table S1.** The Global Physical Activity Questionnaire (GPAQ). The GPAQ measures sedentary behavior and three physical activity domains: work, travel to and from places, and recreational activities. (DOCX 17 kb)

## Data Availability

All data pertaining to the findings are presented in this paper. However, the data can be obtained from the corresponding author any time on reasonable request.

## Abbreviations

aOR: Adjusted Odds Ratio
CI: Confidence Interval
cOR: Crude Odds Ratio
DALY: Disability Adjusted Life Years
DDAC: Dire Dawa Administrative Council
GPAQ: Global Physical Activity Questionnaire
IHRERC: Institutional Health Research Ethics Review Committee
IQR: Inter Quartile Range
LMICs: Low-and Middle-Income Countries
MCAR: Missing completely at random
MET: Metabolic Equivalent Tasks
MICE: Multiple Imputation by Chained Eqs.
NCD: Non-Communicable Diseases
PAF: Population Attributable Fraction
STEPS: WHO STEPwise approach to Surveillance
WHO: World Health Organization
