# Supplementary material for: Level of physical activity among urban adults and the socio-demographic correlates: a population-based cross-sectional study using the global physical activity questionnaire
Source: BMC Public Health. 2019 Aug 22;19:1160. doi: 10.1186/s12889-019-7465-y (PMC6704679; doi:10.1186/s12889-019-7465-y)
Supplement: Supplementary file 1 — Table S1. The Global Physical Activity Questionnaire (GPAQ). The GPAQ measures sedentary behavior and three physical activity domains: work, travel to and from places, and recreational activities. (DOCX 17 kb) [file 12889_2019_7465_MOESM1_ESM.docx]

The Global Physical Activity Questionnaire (GPAQ)

| **Physical Activity** | | |
| --- | --- | --- |
| I am going to ask you about the time you spend doing different types of physical activity in a typical week. Please answer these questions even if you do not consider yourself to be a physically active person. Think first about the time you spend doing work. Think of work as the things that you have to do such as paid or unpaid work, study/training, household chores, harvesting food/crops, fishing or hunting for food, seeking employment. In answering the following questions 'vigorous-intensity activities' are activities that require hard physical effort and cause large increases in breathing or heart rate, 'moderate-intensity activities' are activities that require moderate physical effort and cause small increases in breathing or heart rate. | | |
| **Question** | **Response** | **Code** |
| **Work** | | |
| Does your work involve vigorous-intensity activity that causes large increases in breathing or heart rate like [carrying or lifting heavy loads, digging or construction work] for at least 10 minutes continuously? | Yes 1  No 2 if No, go to P4 | P1 |
| In a typical week, on how many days do you do vigorous intensity activities as part of your work? | Number of days [____] | P2 |
| How much time do you spend doing vigorous-intensity activities at work on a typical day? | Hours: minutes \|_\|_\|:\|_\|_\|  Hrs mins | P3 (a-b) |
| Does your work involve moderate-intensity activity that causes small increases in breathing or heart rate such as brisk walking [or carrying light loads] for at least 10 minutes continuously? | Yes 1  No 2 if No, go to P7 | P4 |
| In a typical week, on how many days do you do moderate intensity activities as part of your work? | Number of days [____] | P5 |
| How much time do you spend doing moderate-intensity activities at work on a typical day? | Hours: minutes \|_\|_\|:\|_\|_\|  Hrs mins | P6 (a-b) |

| **Question** | **Response** | **Code** |
| --- | --- | --- |
| **Travel to and from places** | | |
| The next questions exclude the physical activities at work that you have already mentioned. Now I would like to ask you about the usual way you travel to and from places. For example to work, for shopping, to market, to place of worship. | | |
| Do you walk or use a bicycle (pedal cycle) for at least 10 minutes continuously to get to and from places? | Yes 1  No 2 if No, go to P10 | P7 |
| In a typical week, on how many days do you walk or bicycle for at least 10 minutes continuously to get to and from places? | Number of days [____] | P8 |
| How much time do you spend walking or bicycling for travel on a typical day? | Hours: minutes \|_\|_\|:\|_\|_\|  Hrs mins | P9 (a-b) |
| **Recreational activities** | | |
| The next questions exclude the work and transport activities that you have already mentioned.  Now I would like to ask you about sports, fitness and recreational activities (leisure), [Insert relevant terms]. | | |
| Do you do any vigorous-intensity sports, fitness or recreational (leisure) activities that cause large increases in breathing or heart rate like [running or football] for at least 10 minutes continuously? | Yes 1  No 2 if No, go to P13 | P10 |
| In a typical week, on how many days do you do vigorous intensity sports, fitness or recreational (leisure) activities? | Number of days [____] | P11 |
| How much time do you spend doing vigorous-intensity sports, fitness or recreational activities on a typical day? | Hours: minutes \|_\|_\|:\|_\|_\|  Hrs mins | P12 (a-b) |

| **Question** | **Response** | **Code** |
| --- | --- | --- |
| Do you do any moderate-intensity sports, fitness or recreational (leisure) activities that cause a small increase in breathing or heart rate such as brisk walking, [cycling, swimming, volleyball] for at least 10 minutes continuously? | Yes 1  No 2 if No, go to P16 | P13 |
| In a typical week, on how many days do you do moderate intensity sports, fitness or recreational (leisure) activities? | Number of days [____] | P14 |
| How much time do you spend doing moderate-intensity sports, fitness or recreational (leisure) activities on a typical day? | Hours: minutes \|_\|_\|:\|_\|_\|  Hrs mins | P15 |
| **Sedentary behavior** | | |
| The following question is about sitting or reclining at work, at home, getting to and from places, or with friends including time spent sitting at a desk, sitting with friends, traveling in car, bus, train, reading, playing cards or watching television, but do not include time spent sleeping. | | |
| How much time do you usually spend sitting or reclining on a typical day? | Hours: minutes \|_\|_\|:\|_\|_\|  Hrs mins | P16 (a-b) |
